# Dendritic Cell Therapy in an Allogeneic-Hematopoietic Cell Transplantation Setting: An Effective Strategy toward Better Disease Control?

**DOI:** 10.3389/fimmu.2014.00218

**Published:** 2014-05-19

**Authors:** Maud Plantinga, Colin de Haar, Stefan Nierkens, Jaap Jan Boelens

**Affiliations:** ^1^Utrecht – Dendritic cells AgaiNst CancEr (U-DANCE), Laboratory of Translational Immunology, Department of Immunology, University Medical Centre Utrecht, Utrecht, Netherlands; ^2^Pediatric Blood and Marrow Transplantation Program, Department of Immunology, University Medical Centre Utrecht, Utrecht, Netherlands

**Keywords:** DC-vaccination, hematopoietic cell transplantation, disease control, relapse, T-cell responses

## Abstract

Hematopoietic cell transplantation (HCT) is a last treatment resort and only potentially curative treatment option for several hematological malignancies resistant to chemotherapy. The induction of profound immune regulation after allogeneic HCT is imperative to prevent graft-versus-host reactions and, at the same time, allow protective immune responses against pathogens and against tumor cells. Dendritic cells (DCs) are highly specialized antigen-presenting cells that are essential in regulating this balance and are of major interest as a tool to modulate immune responses in the complex and challenging phase of immune reconstitution early after allo-HCT. This review focuses on the use of DC vaccination to prevent cancer relapses early after allo-HCT. It describes the role of host and donor-DCs, various vaccination strategies, different DC subsets, antigen loading, DC maturation/activation, and injection sites and dose. At last, clinical trials using DC vaccination post-allo-HCT and the future perspectives of DC vaccination in combination with other cancer immunotherapies are discussed.

## Introduction

Allogeneic-hematopoietic (stem) cell transplantation (HCT) is the last treatment resort and only potentially curative treatment option for several hematological malignancies resistant to chemotherapy. Although the survival rates improve after HCT for selected indications, relapses remain a major cause of death after allogeneic HCT. In these high-risk hematological malignancy patients, the estimated 5-year survival rates vary between 10 and 80% (1–3). As such, novel immune therapeutic strategies are being developed aimed at getting better disease control to prevent relapse after HCT.

Currently, the most widely used type of additional immunotherapy combined with allo-HCT is the donor lymphocyte infusion (DLI), where allo-reactive T cells can help to eradicate residual tumor cells. Unfortunately, this “non-specific” strategy suffers from severe toxic side effects, such as Graft-versus-Host Disease (GvHD) (4). Novel immunotherapeutic approaches aim to increase innate or adaptive anti-tumor responses by transferring *ex vivo*-generated effector cells, such as natural killer (NK) cells, chimeric antigen receptor (CAR)-modified cytotoxic T lymphocytes (CTLs), or transgenic T-cell receptor expressing tumor-specific CTLs (5). Although initial results seem promising, the production procedures of these cell therapies are often time-consuming (up to months) and have limitations, severe acute toxicities (“cytokine-release syndrome”: e.g., in CARs), long-term B-cell deficiency (in CD19-CAR), uncertain functionality, and limited or no induction of lasting immunity. Since dendritic cells (DCs) are potent and professional antigen-presenting cells (APC), which induce activation of the adaptive immune system, vaccination strategies could be used post-allo-HCT for the induction of lasting immunity against the tumor. Several vaccination strategies have been used post-allo-HCT like the vaccination with autologous tumor cells either directly transduced to express GM-CSF (6) or coinjected with fibroblast expressing transgenic CD40L and IL-2 (7).

**Figure 1:**
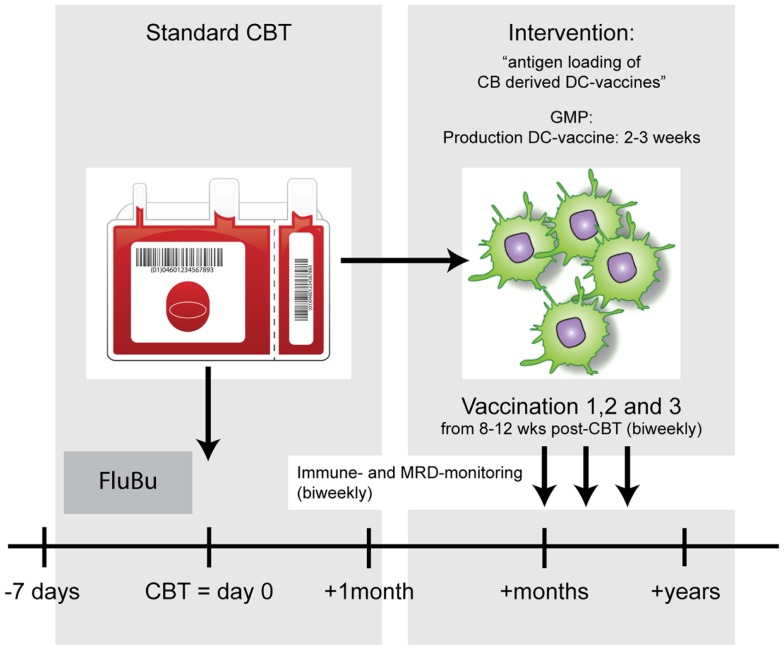
**Example of a DC vaccination strategy to enhance anti-tumor immunity after allo-HCT**. After standard conditioning (FluBu: Fludarabine + Busulfan) and cord blood transplantation (CBT) patients will receive biweekly antigen-loaded-DC vaccines. The timing of vaccinations will be dictated by the chances that most CBT-associated complications are solved or are very unlikely to occur and the T-cell compartment has time to recover.

The use of DCs as vaccines showed beneficial effects in an autologous setting (8), which led to the first FDA approved immunotherapy (9). In this review, we will explore the use of DCs as vaccination strategy for the induction of anti-malignancy responses when combined with an allo-HCT (Figure 1). More specifically, we will focus on the use of donor-derived DCs as part of this immunotherapy.

## Allo-HCT in Cancer Immunotherapy

Allo-HCT is the sole curative option for many patients with high-risk hematologic malignancies and even some solid tumors (4, 10, 11). A variety of different allo-HCT grafts, including bone marrow (BM) or mobilized peripheral blood stem cells (PBSC), as well as unrelated umbilical cord blood (CB) are currently used as a cell source in the treatment of malignancies (12). The therapeutic success of the allo-HCT is not only due to the replacement of the diseased BM but also due to Graft-versus-Leukemia (GvL) or Graft-versus-Tumor (GvT) effects. However, as a trade-off, the potentially life-threatening complication GvHD can occur. In this regard, it is interesting that the use of CB as cell source is associated with lower relapse-rates suggesting stronger GvL-effects, despite lower GvHD-rates compared with BM or peripheral blood as cell source in HCT (2, 13). As such, therapeutic interventions aimed at enhancing the GvT will not necessarily lead to higher rates of GvHD, whereas the active inhibition of GvHD will not necessarily affect the GvT effects (14).

The importance of CTLs in the GvT effects is supported by the observation that an increase in leukemic antigen (WT1) specific CTLs correlated negatively with the WT1 mRNA expression, as a measure of minimal residual disease (MRD) (15). Moreover, the absence of T cells specific for different tumor associated antigen (WT1, MUC1, and proteinase-3) was related to relapses post-allo-HCT in patients with hematological malignancies (16). These data show that tumor-antigen-specific CTLs can be induced after HCT and failure to induce these cells may hamper GvT responses. This strengthens the idea that the active enhancement of tumor-antigen-specific immunity is a viable treatment option to prevent relapses after HCT (17). The development of tumor-antigen-specific CTLs strongly relates to the general immune recovery (especially T cells and DCs) after HCT, a process that is both complex and dynamic, and is affected by a variety of patient and graft-related factors. These include graft source, graft manipulation, age of recipient and donor, conditioning regimen, recovery of thymic output, the occurrence of infections, and GvHD, and their treatment (11, 18–23). Some of these factors will be difficult to control, whereas there are some factors, like the conditioning regimen [especially the serotherapy component: anti-thymocyte globulin (ATG) or Alemtuzumab], which can be more carefully controlled to enhance or get a more predictable immune reconstitution after HCT. In this regard, detailed immune recovery studies showed that the T-cell recovery can be very fast after HCT depending on that timing, dosing, and/or omission of ATG (24). This occurred without causing mayor effects on the development of GvHD [in particular chronic-GvHD (cGvHD)] but with significantly reduced occurrence of viral reactivation, which is strongly dependent on post-HCT T-cell recovery.

A predictable immune reconstitution is of importance to establish an optimal effect of the applied vaccine. Thus vaccination strategies early after allo-HCT, in a setting of a better-predicted immune reconstitution, aiming to prime and/or stimulate tumor-specific CTLs may be an attractive and effective treatment modality.

## DCs and Their Roles in GvHD and GvT Post-Allo-HCT

As professional APCs, DCs have been well recognized for their role in the induction of GvHD on the one hand and GvT responses on the other. Whereas host-derived DCs have shown to be essential for the induction of acute GvHD (aGvHD) in mice, donor-derived DCs intensify aGvHD and may be involved in the development of cGvHD (25, 26). The role of the different DCs in the GvT response after HCT is still poorly understood. From mouse studies, it is known that host DCs may play an important role in GvT responses (27), especially those that are able to cross present tumor-specific antigen (TSA) from tumor cells to the donor T cells (28). The role of host DCs in GvT in humans has been supported in a study where the combination of donor T cells and mixed chimerism in DC subsets induced a potent GvL effect in association with GvHD, whereas DLI in patients with donor chimerism in both T cells and DC subsets resulted in GvL reactivity without GvHD (29).

Largely independent of conditioning regimen and stem cell donor source, a rapid DC chimerism was detected in peripheral blood after allo-HCT (30). Fourteen days after HCT approximately 80% of the DCs were of donor origin increasing up to 95% at 56 days after HCT. With regard to DC chimerism in peripheral tissues, it was found that depending on the regimen, an average 97% of the Langerhans cells (LC) were donor-derived with full intensity conditioning, while 36.5% was donor-derived with reduced intensity conditioning 40 days after allo-HCT. At day 100, at least 90% of the LC was donor-derived (100% in half of the patients) (31). In another study, donor chimerism with median of 95% was detected for LC in skin biopsies taken between day 18 and 56 after HCT (32). However, this same study also indicated that the majority of the patients with an incomplete donor chimerism suffered from aGvHD. Moreover, these data were challenged in a recent paper studying the chimerism in the skin itself, rather than in DCs that migrated from explants (33). This study showed that 3 months after HCT, at least half of the dermal DCs were still of host origin in the absence of aGvHD, suggesting that the mere presence of host DCs is not the cause of aGvHD.

As both host and donor DCs are present after HCT “regular” vaccination strategies (with epitopes from tumor antigens) or targeting DCs *in vivo* as an immunotherapy early after HCT may also be feasible. In patients with a high risk of relapse, the period early after HCT may be crucial for DC-based therapies as the tumor burden is still low and the suppressive immune environment of the tumor can still be overcome. When studies identify a specialized subtype of human DC that may increase GvT without enhancing GvHD, as was shown for CD8α^+^ DCs in mice (28), specific *in vivo* targeting and stimulation of these cells may be a treatment option in the future. Since the *in vivo* targeting of endogenous DC as immunotherapy has recently been extensively reviewed elsewhere this will not be further discussed here (34).

## DC Sources and Subsets for Vaccination in Allo-HCT Setting

Dendritic cells for vaccination purposes can be directly isolated from peripheral blood or can be generated from stem cells residing in the blood or BM. In the post-allo-HCT setting, DCs could be directly isolated from the peripheral blood of the donor. From the blood different subsets can be isolated, namely plasmacytoid DCs (pDCs) and conventional (c)DCs, this latter population can be further subdivided into BDCA1^+^ and BDCA3^+^ DCs. However, the low numbers of in particular circulating into BDCA1^+^ and BDCA3^+^ DCs complicates their clinical application. In an autologous non-HCT setting, promising results were obtained with isolated pDCs. Freshly isolated pDCs that were loaded and activated *ex vivo*, induced antigen-specific CD4^+^ and CD8^+^ T-cell responses in patients suffering from melanoma (35). Despite the low numbers, using DC subsets in current and future trials is relevant and therefore intrinsic properties of DC subsets to stimulate productive T cells should be taken into account in the DC vaccine design.

Dendritic cells may also be generated from precursor cells like CD14^+^ monocytes (from peripheral blood) or CD34^+^ HSC (from BM or peripheral blood), which can be differentiated *ex vivo* into monocyte-derived DCs (moDCs) or conventional DCs, respectively.

After the finding that monocytes develop DC-like features when cultured in the presence of GM-CSF and IL-4 (36), moDCs have been used in many clinical trials as a cancer immunotherapy. The use of moDCs as a vaccine is generally considered as safe, but clinical responses have only sporadically been observed (37), possibly due to maturation status or migratory capacity, discussed in more detail below. Since more research focuses on differential functionalities within DC subsets, the vaccine research shifts toward targeting of specialized DC subsets (34, 38) and *in vitro* generation of conventional DCs from CD34^+^ precursor stem cells. Several protocols have been developed trying to mimic the different naturally occurring DC-populations (39–41), so far no clinical data are available on the efficacy of these DC cultures. The most important advantage of using CD34-derived DC, especially in the CB HCT setting, is the possibility to use an expansion step prior to DC differentiation allowing the generation a large number of DCs from a limited number of precursor cells.

Although studies directly comparing the anti-leukemic effects of CD14- versus CD34-derived DC vaccines are lacking (42), it has been suggested that CD34-derived DCs may induce better CD8 responses, compared to moDCs. This might be caused by the presence of LC in these cultures (43). The presence of LC is however strongly dependent on the presence of specific growth factors during differentiation.

## DC Vaccination Strategies

**Figure 2:**
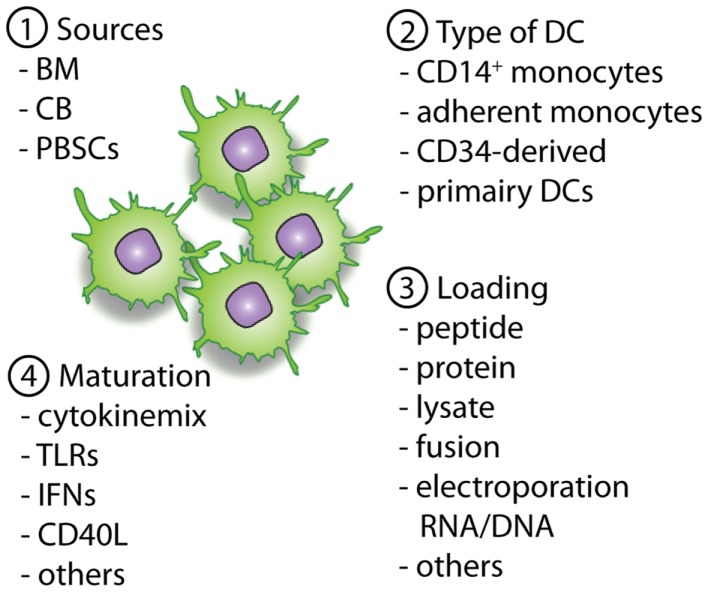
**Overview of important parameters to consider and optimize pre-clinically with regard to DC vaccines**. The first important parameter is the source of the allo-HCT graft (1), which will determine the available cell sources for the generation of the DCs (2). When DCs are generated the antigen loading strategy (3) will define the presentation of (tumor)-antigens in MHC-class II and I molecules, providing the first signal for T-cell activation. Next, optimal maturation signals should be used to induce the expression of co-stimulatory molecules and the necessary cytokines (signal 2 and 3). This will enable homing of the DCs to lymph nodes followed by an optimal stimulation of antigen-specific T cells for the induction of lasting immunity.

Besides the type of DC, the specific antigen loading and maturations strategies have major impact on the priming capacity of the DC. In addition, the functionality of the DC vaccine is dependent on the infection site, dosing regimen, and timing of vaccination, all of which may be even more prominent when combined with allo-HCT (Figure 2).

### Tumor-antigen loading

Different loading strategies have been developed over the years, reviewed by Nierkens et al. (44). Exogenous MHC-class I loading with a 9-mer peptide of a pre-defined tumor antigen is frequently used. Although the analyses of T-cell specificity against that one peptide may simplify immune monitoring, this system has however some major disadvantages, such as, HLA-restriction, epitope spreading by the tumor, and lack of induction of antigen-specific CD4 T cells. Alternatively, DCs can be loaded with long-peptides, containing several MHC-class I and II restricted tumor-antigen peptides, 15-mer peptide pools covering the whole tumor antigen, or the whole tumor antigen (protein or mRNA). These approaches require the prior identification of the TSA. For several tumors specific antigens may however not be known. In these cases, whole tumor cell lysates, DC-tumor cell fusions, or apoptotic/necrotic tumor cells can be used as a source of tumor antigens (45, 46). Although vaccination with tumor cells or their lysates may induce/aggravate acute or cGvHD due to the presentation of allo-antigens shared by tumor and normal host cells, to date, none of the studies using tumor cells as part of their vaccine showed any induction of exacerbation of GvHD (6, 7, 47, 48). As such, loading DC vaccine with killed tumor cells or lysates may be an attractive alternative when specific tumor antigens are not known, when they differ between the patients with the same cancer or when the proteins are sensitive to mutations.

### Maturation

For the stimulation of antigen-specific T cells DCs require maturation, which can be induced by clinical grade maturation mixes. Classically, moDCs are matured with a mix of pro-inflammatory cytokines, e.g., IL-1b, IL-6, TNF, and PGE2, which induce strong upregulation of CD40, CD80, CD83, CD86, and CCR7 (49, 50) and are clinical grade available. PGE2 has been shown to be necessary for the migration of DCs (51), but it also induces IDO expression (52), which is involved in inducing tolerogenic responses. However, Krause et al. showed IDO expression independently of PGE2, and strong CD4 and CD8 proliferation after co-stimulation with DCs matured with PGE2, despite IDO expression (53).

Dendritic cells also express different pathogen recognition receptors, like Toll-like receptors (TLRs). Although TLR antagonists have been shown to be good candidates for DC activation, their use as maturation agent in DC vaccination trials is still limited. Currently, PAM3cys for TLR2, Poly-IC or Poly-ICLC (Hiltonol) for TLR3, LPS for TLR4, or Imiquimod TLR7 and CpG-ODN for TLR9 are clinical grade available and used in several combinations (with each other or with cytokines) in clinical trials (54–56). The effect of the maturation mixes strongly depends on the DC subset isolated or cultured, since different DC subsets express different TLRs (57). In several clinical trials, the combination of TLR agonists or cytomix is used with IFN type 1 or 2, TNF, or CD40L (58–61). This combination not only enhances their maturation efficacy, but also induces stronger cytokine production *in vitro* (59). CD40L is used to activate DCs *in vitro* before injection, and although DC maturation and IL-12 production was reported, no clinical benefit was observed (62). One could even speculate that stimulation with CD40L before the vaccination infusion may somehow activate the DC before they were able to connect with the antigen-specific T cells in the lymph nodes.

In addition to co-stimulatory molecules, DCs are also known to express co-inhibitory molecules, like PD-L1 and PD-L2, which may hamper T-cell stimulation via interaction with PD1. Targeting the expression PD-L1 and PD-L2 siRNA electroporation or transfection into DCs has been shown to enhance CTL responses *in vitro* and *in vivo* (63, 64). Since this approach can be incorporated into DC vaccines relatively easy, this has the potential to become a standard procedure in addition to the maturation for future DC vaccinations.

### Injection sites and dosing and timing

When a DC vaccine is optimally loaded and matured, the next border to cross is to consider the optimal injection site. In clinical vaccination studies, DCs have been injected intravenously (i.v.), intradermal (i.d.), subcutaneously (s.c.), directly in the lymph node (i.n.) under sonographic guidance, or intratumoral (i.t.) or at different sites within the same trial. Side to side comparisons of injection sites are generally lacking making it hard to make a strong statement on which site would be preferable. Intratumoral DC vaccination has been shown to be safe (65). The question remains whether the DCs are needed at the tumor site to restimulate the tumor infiltrating lymphocytes (TILs) or that they are required to present their cargo in the lymph node for the priming of novel CTLs, in which case other sites of injection could be a better option. Furthermore, the strong immune suppressive environment in the tumor may be detrimental for CTL activation. Bedrosian et al. (66) showed in a phase I trial in metastatic melanoma patients that i.n. is superior over i.d. with regard to CTL induction. Whereas the study of Kyte et al. showed no advantage of injecting i.d. compared to i.n. in a phase I/II trial also in melanoma patients (67). The type of DC used for vaccination or disease stage could both contribute to these contradictory findings. The limited overall efficacy of DC vaccination may further hamper the proper comparison between the different injection sites.

Another variation within clinical trials is the frequency and dosing regimen, varying from 2 to 6 times. No clear comparison has been made, and therefore no strong conclusions can be drawn. According to mouse studies and some clinical trials, vaccination seems to be critical, but boosting strategies of subjects with residual disease or with tumor recurrence, should be carefully revisited (68).

## Monitoring the Effect of DC Vaccination

Over 1000 trials have been performed using DC vaccination, but read-outs are very diverse, and mainly phase I/II trials test for cytotoxicity and overall survival are studied. The immunological CTL response generated by the DC vaccination can be monitored using HLA-peptide tetramers or by assessing cytokine production after *ex vivo* antigen-specific restimulation (ELISA, ELISPOT, or intracellular flow cytometry).

Since most DC vaccinations have been performed in an autologous (HCT) setting there may be tumor-antigen-specific T cells present. To be able to differentiate between priming and reactivation of T cells, KLH is sometimes used as a reporter for the presence of priming and Influenza Matrix Protein (Flu-MP) could be added as positive control for reactivation. When combined with peptide-loaded DCs, these proteins may also be helpful in providing bystander CD4 help (69). Almost all patients receiving DC vaccination in the skin are tested at several time points after vaccination for a delayed type hypersensitivity (DTH) response however most of these responses are KLH or Flu-MP specific and might not necessarily be predictive of the induced anti-tumor responses (70).

With regard to tetramer staining to study antigen-specific CTLs, the recent development of conditional HLA-ligand peptide exchange technology combined with combinatorial coding may provide an excellent opportunity to check for a wide range of different peptide–HLA combination in limited amount of material (71, 72).

With increasing sensitivity of PCR techniques, MRD markers are increasingly used to monitor clinical efficacy of immune therapy (73), including DC vaccination (74). A more general approach is immune-phenotyping analysis for the frequency of different immune cells at several time points before and after vaccination. These kind of analysis have reported changes in NK cells and their activation status after DC vaccination (74). Since current DC vaccines are still limited in their potential to induce an effective anti-tumor immune response, the possibility to compare results from different studies could benefit from “international standardized” immuno-monitoring protocols (75).

## DC Vaccination Trial in Allo-HCT

Although the use of DC vaccination after allo-HCT had been suggested for many years, Grigoleit and colleagues were the first to publish a phase 1/2 clinical trial using donor CD14-derived DC after HCT in patients at high risk for developing CMV disease (76) (Table 1). In this setting, peptide-loaded DCs were injected s.c. near the inguinal lymph node. Immune monitoring showed the induction of CMV-specific T-cell responses, which had clinical effects on CMV disease in a prophylactic as well as therapeutic setting. With regard to the potential adverse events, it was important to notice that vaccination with donor-derived DCs pulsed with HCMV peptides did not stimulate or expand allo-reactive T cells. Nor were there any long-term adverse effects of DC vaccination after HCT. Taken together, this phase 1/2 study provided the first evidence indicating that DC vaccination can be performed safely in allogeneic HCT setting. DC vaccination was also performed in a therapeutic setting in a patient suffering from recurrent CMV reactivation after a second HCT (77). As there was emerging viral resistance to the antiviral chemotherapy, DC cells were prepared from CD14^+^ monocytes isolated from the patients PB and loaded with CMV PP65 protein. The induction of PP65 specific CD4 and CD8 cells was detected and coincided with lasting prevention of CMV recurrence. This study is strongly supportive of the use of protein instead of peptide to enable the induction of both CD4 and CD8 responses. In this study again no adverse events were reported.

**Table 1:** **Overview of DC vaccination trials after allo-HCT**.

Source stem cells	Source DC	(Tumor) target	Antigen	Antigen form	Vaccination	Read-out	Immune response	Clinical response	(S)AE	Reference
BM	PBSC	AML aLL	Whole tumor	Apoptotic tumor cells	IV	Vitro CTL/MLR DTH	DTH 3/4	3/4	NR	(47)
PBSC	CD14+	Renal cell carcinoma	Autologous tumor	Lysate	ID	DTH	0/1	0/1	NR	(48)
BM/PBSC	CD14+	CMV	Pp65 pp150	Peptide	SC near LN	Tetramer peptide recall	7/17 (41%)	YES link IR?	NR	(76)
BM/PBSC?	CD14+	AML	WT1 KLH reporter	Peptide	ID (6 month after HCT)	Tetramer peptide recall	KLH yes WT1 no	0/1	NR	(78)
BM/PBSC?	CD14+	CMV	PP65	Protein	SC near ILN (6 month after second HCT)	Protein recall	1/1	1/1	NR	(77)
BM/PBSC?	CD14+ host-derived	MM	Allo-antigens MiHA KLH reporter	Protein	ID near ILN (6 month after second HCT)	Protein recall DTH	KLH 6/6	No but patients also did not respond to DLI	NR	(4)

*(S)AE, (Severe) adverse events*.

The first publication using DCs to boost the GvT responses after HCT was by Fujii and colleagues (47). Four patients with hematological malignancies relapsed after allo-HCT and were treated with DCs cultured from PBSC isolated from the same donor as the HCT. These donor-derived DCs were then loaded with tumor cells from the patient that were induced to go into apoptosis by irradiation. DCs were then injected i.v. and clinical response was reported in three out of four patients characterized by the reduction in tumor load. No side effects were detected in any of the patients. In a following case report, DC vaccination was used in a patient who received an allo-PB-HCT as a treatment for renal cell carcinoma (48). However in this patient no antigen-specific recall response (DTH) or any clinical response was reported. Like the previous report, this patient also did not show any severe adverse events.

Another case report describes vaccination with CD14-derived DCs pulsed with WT1 peptide and KLH antigen for the treatment of AML relapse after allo-HCT (78). Although no WT1 peptide-specific T cells could be detected, the KLH specific DTH and ELISPOT further support the ability of DC vaccination to induce an antigen-specific immune response in a patient after allo-HCT. Host CD14-derived DCs, isolated prior to allo-HCT, were used to present minor histocompatibility antigens (MiHA) antigens in six multiple myeloma patients that had received auto-HCT followed by allo-HCT and two rounds of DLI (4). This study showed that DC vaccination using host-MoDCs was safe (no GvHD) when applied at least 6 months after HCT induced immunity (KLH). Unfortunately, no MiHA specific T cells were detected after vaccination and also clinical responses were poor, probably caused by the setup of the treatment protocol.

So, although only very limited studies have been reported using DC vaccination after allo-HCT, the data so far are promising with some clinical responses, detectable immune responses, and no increase in the adverse events normally occurring after allo-HCT, all ruling in favor of further exploration of DC vaccination in allo-HCT. In additional, ongoing or recently finished, trials patients are treated utilizing idiotype-pulsed allogeneic DCs post-allo-HCT (NCT00186316 clinicaltrials.gov) or with donor-derived DCs pulsed with WT1 peptides in combination with DLI (NCT00923910 clinicaltrials.gov).

## Combination Therapies

The limited clinical efficacy of DC vaccination may not only be due to the vaccine or vaccination strategy since the final eradication of the tumor depends on a variety of factors within the cancer-immunity cycle (79). When antigen-specific CTLs are induced and go to the tumor site there are mechanisms in place that prevent the tumor cells from getting killed by CTLs, i.e., downregulation of activation receptors, co-stimulatory molecules, or HLA class I antigens recognized by CTLs; upregulation of co-inhibitory molecules like PD-L1 release of soluble factors that inhibit Th cells, CTLs, and APCs; and altered FAS-L expression on the tumor cells causing apoptosis resistance (80–83). Clinical trials with therapies aimed at these immune blockades, such as cytotoxic t-lymphocyte-associated antigen 4 (CTLA4) and programed cell death protein 1 (PD1), have shown some very promising results as reviewed recently (84), making some of the therapies interesting candidates to use in combination with DC vaccination. This is supported by the observation in combination with a DC vaccine, a PD1 blocking antibody enhances *ex vivo* activated T-cell responses after DC/tumor fusion stimulation (85).

Another post-HCT immune therapy that can be combined with DC vaccination is the infusion of tumor antigen or MiHA specific CTLs that can provide additional effector cells to reduce the tumor burden if disease has relapsed. In this way, it may also affect the tumor microenvironment enabling better migration and CTL function of the DC generated CTLs. The use of PBSC or BM as HCT graft has the obvious advantage that DLI can be performed as a prophylaxis or therapy combined with DC vaccination (4, 86, 87). Another possibility is the use TCR gene transfer for the formation of a large population of tumor-antigen-specific T cells that would reduce the risk of GvHD or other bystander immune responses (88, 89). All these latter techniques remain to be tested in combination with DC vaccination.

Very recently, epigenetic drugs were used in combination with DC vaccination to enhance MHC upregulation, and therefore tumor-antigen expression on the tumor cells. A very promising clinical trial in a stage IV Neuroblastoma (NB) patient showed complete remission with this combined therapy (90).

To take DC vaccination to the next level one should consider making use of these additional therapies to hopefully enhance clinical efficacy of DC vaccination in all immune therapeutic settings.

## Summary

Although allogeneic-hematopoietic (stem) cell transplantation (HCT) is the only potentially curative treatment option for several hematological malignancies resistant to chemotherapy, relapses remain a major problem. DC vaccination may be an attractive additional immune therapeutic option for the induction of specific anti-malignancy immune responses in the context of an allo-HCT setting. Factors like optimizing and predicting immune recovery suggest that a more personalized conditioning regimen especially considering the use of ATG is essential for optimal effect of the vaccine. Depending on the HCT graft source different DC sources can be considered, with currently no conclusive data on which source to prefer. Preclinical development of the DC vaccine should further contain the optimization of antigen loading, DC maturation as well as limitation of the expression of co-inhibitory molecules. Finally, one should carefully consider the injection site and dose and frequency of the DC vaccine. The few DC vaccinations studies after allo-HCT have shown to be safe as well as promising with regard to both clinical and immunological responses. As such the field is open for further exploration especially with the current advances in possible combination therapies to further reduce the relapse rates and improve the survival rates.

## Conflict of Interest Statement

The authors declare that the research was conducted in the absence of any commercial or financial relationships that could be construed as a potential conflict of interest.
